# Tandem UIMs confer Lys48 ubiquitin chain substrate preference to deubiquitinase USP25

**DOI:** 10.1038/srep45037

**Published:** 2017-03-22

**Authors:** Kohei Kawaguchi, Kazune Uo, Toshiaki Tanaka, Masayuki Komada

**Affiliations:** 1 Cell Biology Unit, Institute of Innovative Research, Tokyo Institute of Technology, Yokohama 226-8501, Japan; 2 School of Life Science and Technology, Tokyo Institute of Technology, Yokohama 226-8501, Japan

## Abstract

Ubiquitin-specific protease (USP) 25, belonging to the USP family of deubiquitinases, harbors two tandem ubiquitin-interacting motifs (UIMs), a ~20-amino-acid α-helical stretch that binds to ubiquitin. However, the role of the UIMs in USP25 remains unclear. Here we show that the tandem UIM region binds to Lys48-, but not Lys63-, linked ubiquitin chains, where the two UIMs played a critical and cooperative role. Purified USP25 exhibited higher ubiquitin isopeptidase activity to Lys48-, than to Lys63-, linked ubiquitin chains. Mutations that disrupted the ubiquitin-binding ability of the tandem UIMs resulted in a reduced ubiquitin isopeptidase activity of USP25, suggesting a role for the UIMs in exerting the full catalytic activity of USP25. Moreover, when mutations that convert the binding preference from Lys48- to Lys63-linked ubiquitin chains were introduced into the tandem UIM region, the USP25 mutants acquired elevated and reduced isopeptidase activity toward Lys63- and Lys48-linked ubiquitin chains, respectively. These results suggested that the binding preference of the tandem UIMs toward Lys48-linked ubiquitin chains contributes not only to the full catalytic activity but also to the ubiquitin chain substrate preference of USP25, possibly by selectively holding the Lys48-linked ubiquitin chain substrates in the proximity of the catalytic core.

Ubiquitin is a 76-amino-acid protein highly conserved in eukaryotes. It is conjugated through its C-terminal carboxyl group to the ε-amino group of Lys residues of numerous intracellular proteins by an isopeptide bond. This posttranslational modification is catalyzed by sequential actions of E1 (ubiquitin-activating), E2 (ubiquitin-conjugating), and E3 (ubiquitin ligase) enzymes. Ubiquitin can also be conjugated to one of the seven internal Lys residues or the N-terminal α-amino group of another ubiquitin, allowing the formation of poly-ubiquitin chains on the substrate proteins^1^,^2^. Poly-ubiquitin chains have different tertiary structures depending on which of the eight amino groups is used to elongate the chain, and these different types of polyubiquitination serve as different cellular signals^1^,^2^. For instance, Lys48-linked polyubiquitination is a well-known tag which directs the modified proteins to the proteasome for degradation. Lys63-linked ubiquitination regulates multiple cellular events including DNA repair, signal transduction, and lysosomal traffic of plasma membrane proteins.

Ubiquitination is a reversible modification opposed by deubiquitinases (DUBs) which hydrolyze the isopeptide bond between ubiquitin and substrate proteins as well as between ubiquitins in ubiquitin chains. The human genome encodes ~90 DUBs, and based on the amino acid sequences of the catalytic domains, they are classified to five families: USP (ubiquitin-specific protease), UCH (ubiquitin C-terminal hydrolase), OTU (ovarian tumor-related protease), ataxin-3/Josephin, and JAMM (Jab1/MPN/Mov34)^3^,^4^. While the JAMM DUBs are metalloproteases, DUBs in the other four families are Cys proteases. Ubiquitination-dependent regulation of protein functions is balanced by E1/E2/E3-mediated ubiquitination and DUB-mediated deubiquitination. As different linkage types of polyubiquitination regulate the function of target proteins in different ways, the isopeptidase specificity of DUBs toward different linkages of ubiquitin chains is potentially important to precisely control the protein activities in the cell. Indeed, some of the DUBs selectively or preferentially disassemble ubiquitin chains with specific linkages^5^,^6^,^7^,^8^. While underlying mechanisms for the substrate specificity toward Lys63-linked ubiquitin chains have been elucidated for several DUBs^5^,^6^, those toward Lys48-linked chains, the most abundant chain type in the cell, are unknown.

Tumor necrosis factor receptor-associated factor 3 (TRAF3) is an E3 ubiquitin ligase which regulates the adaptive as well as innate immune signaling downstream of various cytokine and pattern recognition receptors. The signaling activity of TRAF3 is differentially regulated by different chain types of polyubiquitination^9^. Lys48-linked polyubiquitination, mediated by the E3 ligase cIAP, triggers the proteasomal degradation of TRAF3, which releases receptor-associated MEKK1 to the cytoplasm and activates the MAP kinase pathway, leading to the production of pro-inflammatory cytokines^10^. In contrast, Lys63-linked polyubiquitination of TRAF3, mediated by the E3 activity of TRAF3 itself, activates TBK1 and IKKε protein kinases, leading to phosphorylation-dependent activation of the transcription factor IRF3 and expression of type I interferons^11^. Therefore, if ubiquitination-mediated regulation of the TRAF3 activity is balanced by deubiquitination, the responsible DUBs need to have a substrate specificity toward Lys63- or Lys48-linked ubiquitin chains in order to suppress a single signaling pathway at a time. Indeed, DUBA/OTUD5, a DUB which selectively disassembles Lys63-linked ubiquitin chains on TRAF3, has been shown to suppress the production of type I interferon by reducing the level of TRAF3-TBK1 interaction^12^. Other DUBs, such as OTUB1/2^13^,^14^ and UCHL1^15^, are also reported to suppress the type I interferon production pathway by removing Lys63 ubiquitin chains from TRAF3.

On the other hand, USP25, a DUB of the USP family, has been shown to inactivate the TRAF3-mediated MAP kinase signaling by selectively removing Lys48-linked ubiquitin chains from TRAF3 and thereby preventing its proteasomal degradation^16^,^17^. In addition to the catalytic core composed of the Cys- and His-boxes conserved in the USP family of DUBs, USP25 contains three potential ubiquitin-binding domain/motifs in the N-terminal region; the ubiquitin-associated (UBA) domain followed by two tandem UIMs. The UIM is a ~20-amino-acid ubiquitin-binding α-helical stretch with a consensus sequence of -AcAcAc-Φ--AlaΦ--Ser--Ac-, where Ac and Φ represent acidic (Asp or Glu) and bulky hydrophobic (Leu or Ile) residues, respectively^18^,^19^. In this study, we provide evidence for UIM-mediated regulation of the substrate preference of USP25 toward Lys48-linked ubiquitin chains, which, for the first time, addresses the mechanistic basis of Lys48 ubiquitin chain substrate selectivity of DUBs.

## Results

### Construction of USP25 mutants lacking functional UIMs

**Figure 1:**
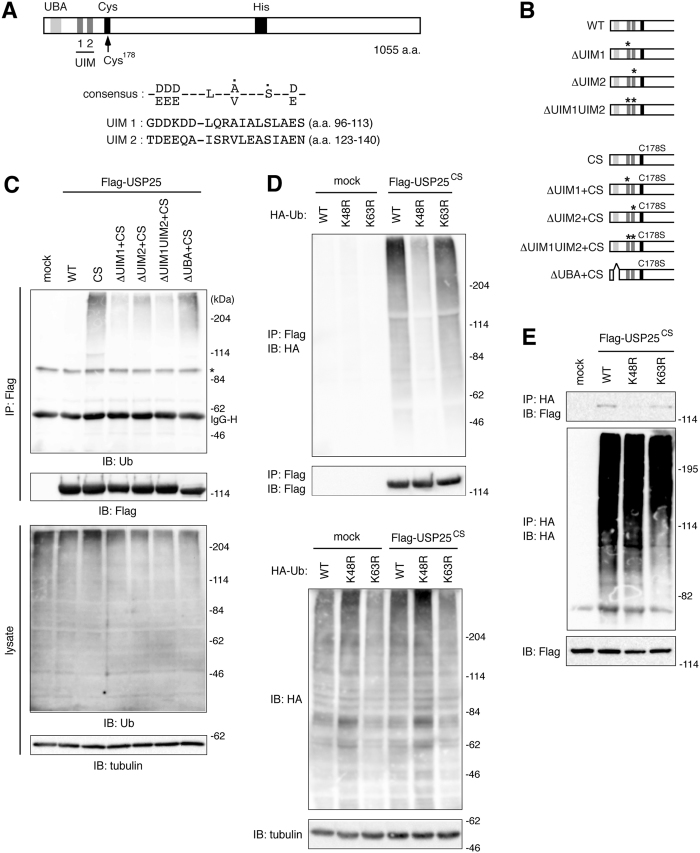
UIM-dependent USP25 binding to endogenous ubiquitin-protein conjugates. (**A**) A schematic domain structure of human USP25 and the sequence alignment of UIM1 and UIM2, together with the consensus UIM sequence, are shown. The Ala/Val and Ser residues indicated with a dot in the consensus sequence were replaced by Gly and Ala, respectively, in the mutants shown in (**B**). (**B**) USP25 mutants lacking functional UIMs, UBA domain, and catalytic activity, used in this study are shown. Asterisks indicate the point mutations in UIMs introduced in each mutant. CS indicates a replacement of Cys^178^ to Ser in the Cys-box of the catalytic core. (**C**) HEK293T cells were transfected with the indicated Flag-USP25 constructs. Their lysates were immunoprecipitated (IP) with anti-Flag antibody, and immunoblotted (IB) with anti-ubiquitin (Ub) and anti-Flag antibodies. IgG-H: IgG heavy chain used for immunoprecipitation; asterisk: non-specific band. (**D**,**E**) Lysates of HEK293T cells transfected with HA-tagged ubiquitin^K48R^ (K48R) or ubiquitin^K63R^ (K63R) were mixed with the lysate of HEK293T cells transfected with Flag-USP25^CS^. The mixed lysates were immunoprecipitated (IP) with anti-Flag (**D**) or anti-HA (**E**) antibody, and immunoblotted (IB) with indicated antibodies.

Amino acid sequences of the two tandem UIMs in human USP25, together with the consensus UIM sequence, are aligned in Fig. 1A. To study the role of the UIMs in USP25, designated as UIM1 and UIM2 from the N-terminal side, we introduced point mutations into each UIM individually (Fig. 1B, ΔUIM1 and ΔUIM2) or in combination (Fig. 1B, ΔUIM1UIM2). In the mutants, the invariant Ala/Val and Ser residues (Fig. 1A, indicated with a dot in the consensus sequence) in each UIM were replaced by Gly and Ala, respectively, because this combination of mutations has been shown to fully abolish the ubiquitin-binding ability of UIMs in other proteins^20^,^21^,^22^. We further introduced a catalytically-inactivating point mutation CS, which replaces the Cys178 residue in the Cys-box of the catalytic core with Ser, to wild-type (WT) as well as the above-mentioned mutants of USP25 (Fig. 1B). We also constructed a C178S mutant deleted of the whole UBA domain (Fig. 1B).

### Tandem UIMs of USP25 bind to endogenous ubiquitin-protein conjugates in the cell

We first examined the ubiquitin-binding ability of the WT and UIM mutants of USP25 in the cell by detecting endogenous ubiquitin-protein conjugates co-immunoprecipitated with transfected Flag epitope-tagged USP25 constructs. To prevent the USP25-catalyzed hydrolytic removal of conjugated ubiquitins from USP25-associated ubiquitinated proteins, we used the USP25 constructs which also harbor the catalytically-inactivating CS mutation. The ΔUIM+CS mutants were transfected to HEK293T cells and immunoprecipitated from their lysates with anti-Flag antibody. Immunoblotting of the precipitates with anti-ubiquitin antibody demonstrated that USP25^CS^ co-precipitates endogenous ubiquitin-protein conjugates (Fig. 1C). The amount of the ubiquitin-protein conjugates co-precipitated with USP25^ΔUIM1+CS^ or USP25^ΔUIM2+CS^ was significantly reduced (Fig. 1C). In particular, the ability of USP25^ΔUIM1+CS^ was as weak as USP25^ΔUIM1UIM2+CS^ with mutations in both UIM1 and UIM2. Quantification of the amounts of co-precipitated ubiquitin-protein conjugates suggested that the effect of the tandem UIMs on ubiquitin binding is cooperative rather than additive (Supplementary Fig. S1A). In contrast, deletion of the UBA domain did not significantly affect USP25 binding to endogenous ubiquitin-protein conjugates (Fig. 1C, USP25^ΔUBA+CS^).

### Tandem UIMs of USP25 preferentially bind to Lys48-linked ubiquitin chains

To study the chain specificity of ubiquitin binding by USP25, we expressed HA epitope-tagged K48R (Lys48 replaced by Arg) and K63R (Lys63 replaced by Arg) mutants of ubiquitin, which block the formation of Lys48- and Lys63-linked ubiquitin chains, respectively, in HEK293T cells. After mixing the lysates of these cells with the lysate of HEK293T cells transfected with Flag-USP25^CS^, Flag-USP25^CS^ was immunoprecipitated with anti-Flag antibody, and co-precipitated cellular ubiquitin-protein conjugates were detected by immunoblotting with anti-HA antibody. While USP25^CS^ successfully pulled down HA-tagged ubiquitin-protein conjugates from the lysate of WT HA-ubiquitin-transfected cells, the amount of co-precipitated ubiquitinated proteins was significantly reduced when the formation of Lys48-linked ubiquitin chains was blocked by ubiquitin^K48R^ expression (Fig. 1D, see Supplementary Fig. S1B for quantification). In contrast, blocking the formation of Lys63-linked ubiquitin chains with ubiquitin^K63R^ did not affect the amount of ubiquitinated proteins co-precipitated with USP25^CS^ (Fig. 1D). Consistently, in a converse experiment, ubiquitinated proteins co-immunoprecipitated Flag-USP25^CS^ from WT and K63R, but not K48R, ubiquitin-expressing cells (Fig. 1E, Supplementary Fig. S1C), suggesting that USP25 exhibits binding preference to Lys48-linked ubiquitin chains.

**Figure 2:**
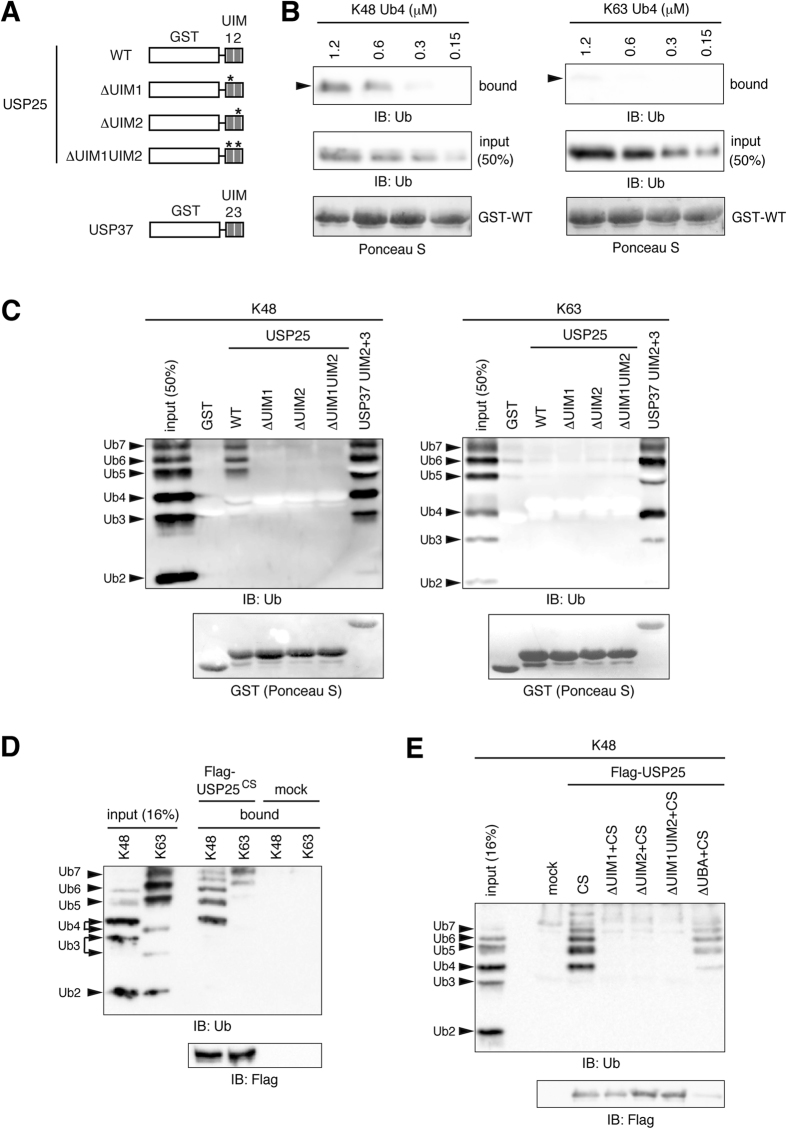
Ubiquitin chain binding features of UIMs in USP25. (**A**) Schematic structures of GST-USP25 UIM fusion proteins used in (**B**,**C**) are shown. The GST-USP37 UIM fusion protein was used as a positive control. (**B**,**C**) Indicated GST-UIM fusion proteins illustrated in (**A**) were immobilized on glutathione beads and incubated with indicated concentrations of Lys48 (K48)- or Lys63 (K63)-linked tetra-ubiquitin (**B**) or ubiquitin oligomers (mixture of dimer-heptamer) (**C**). After washing the beads, bound ubiquitin chains were detected by immunoblotting with anti-ubiquitin antibody (top). The amounts of the GST-fusion protein used in the experiments were assessed by staining with Ponceau S (bottom). (**D**,**E**) Indicated Flag-tagged USP25 proteins were immunopurified from the “hot-lysis” lysates of transfected HEK293T cells, immobilized on anti-Flag antibody-conjugated beads, and incubated with Lys48 (K48)- or Lys63 (K63)-linked ubiquitin oligomers (mixture of dimer-heptamer). After washing the beads, bound ubiquitin chains were detected by immunoblotting with anti-ubiquitin antibody (top). The amounts of Flag-USP25 proteins used in the experiments were assessed by anti-Flag immunoblotting (bottom).

To examine the ubiquitin-binding characteristics of purified USP25, we first expressed the region of USP25 harboring the tandem UIMs as a glutathione *S*-transferase (GST)-fusion protein in *E. coli* cells (Fig. 2A, GST-WT). We also introduced the same point mutations as in Fig. 1B to the GST-fusion protein (Fig. 2A, GST-ΔUIM1, -ΔUIM2, and -ΔUIM1UIM2). When different concentrations of Lys48- or Lys63-linked tetra-ubiquitin chain was incubated with the WT UIM region, Lys48 chain was clearly, while Lys63 chain was hardly, pulled down (Fig. 2B, Supplementary Fig. S1D). We next examined the binding of GST-fused WT as well as the mutant UIM regions to Lys48- and Lys63-linked ubiquitin oligomers (mixture of dimer-heptamer). Detection of ubiquitin oligomers that were pulled down with the GST-fusion proteins using anti-ubiquitin antibody showed that the WT UIM region binds to Lys48-, but not Lys63-, linked ubiquitin chains (Fig. 2C, Supplementary Fig. S1E). As a control, the UIM region of USP37 equally pulled down Lys48- and Lys63-linked ubiquitin chains as reported^22^. Introduction of mutations into either UIM1 or UIM2 led to almost complete loss of binding to Lys48 ubiquitin chains, indicating that the tandem UIMs play a cooperative role in ubiquitin binding.

To exclude the possible artificial effect of GST dimerization on ubiquitin binding of the GST-fused UIM region, we also purified Flag-tagged full-length USP25^CS^ from transfected HEK293T cells. To strip cellular ubiquitin-protein conjugates bound to the tandem UIMs of USP25, Flag-USP25^CS^-transfected cells were lysed in boiling 1% sodium dodecyl sulfate (SDS) solution (hot-lysis) and immunoprecipitated with anti-Flag antibody after renaturation. Pull-down experiments using these immunopurified USP25 proteins showed that WT USP25 binds preferentially to Lys48-linked ubiquitin chains (Fig. 2D, Supplementary Fig. S1F). In addition, mutations in either UIM1 or UIM2 significantly reduced the ubiquitin-binding ability of full-length USP25 (Fig. 2E, Supplementary Fig. S1G). As a common feature of UIMs in other proteins^23^, the UIM region of USP25 exhibited much higher affinity to long ubiquitin oligomers (tetramer-heptamer) than to mono-, di-, or tri-ubiquitin (Fig. 2D,E). Collectively, the results in Figs 1 and 2 suggested that USP25 is a Lys48-linked chain-preferring ubiquitin-binding protein in which the tandem UIMs play a central and cooperative role.

### USP25 exhibits isopeptidase activity preferentially toward Lys48-linked ubiquitin chains

**Figure 3:**
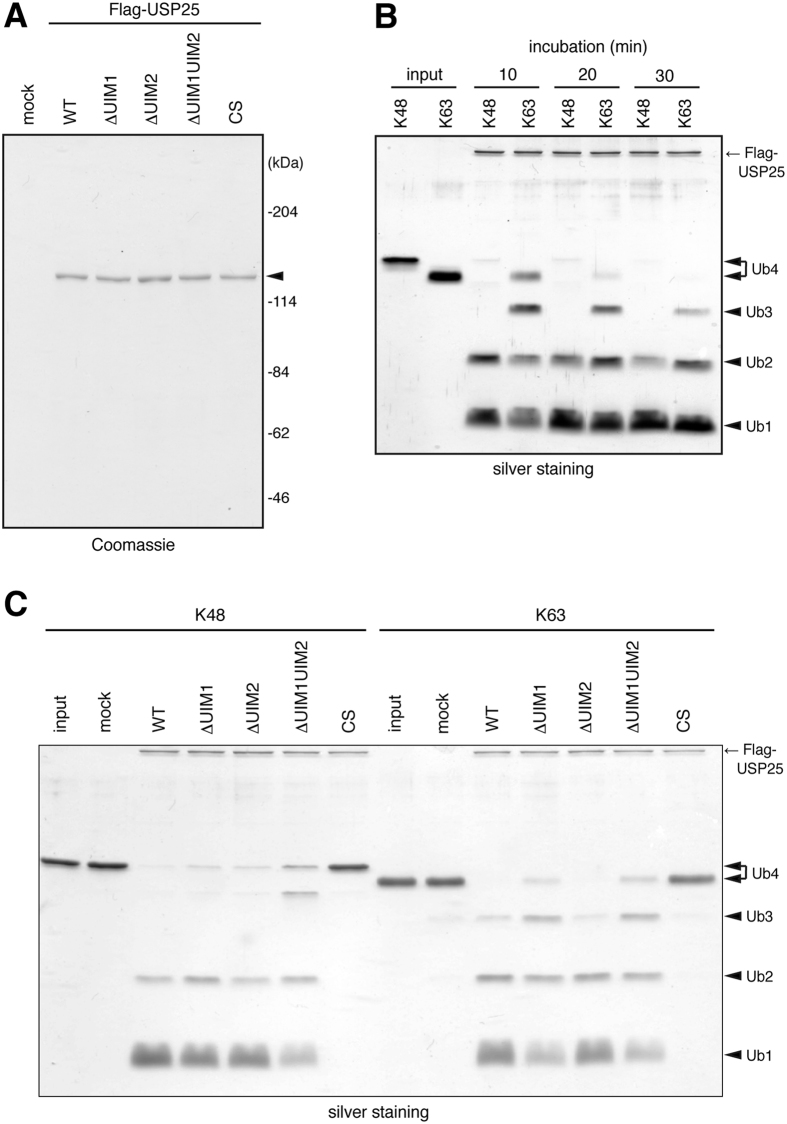
Ubiquitin chain substrate preference and UIM dependence of USP25 catalytic activity. (**A**) Indicated Flag-tagged USP25 constructs were expressed in HEK293T cells, immunoprecipitated from their lysates with anti-Flag antibody, and eluted from the antibody with the competing Flag peptide. Eluted proteins were detected by Coomassie staining after SDS-PAGE. An arrowhead indicates the USP25 proteins. (**B**,**C**) WT USP25 (**B**) and its mutants (**C**), prepared in (**A**), were incubated with Lys48- and Lys63- linked tetra-ubiquitin (Ub4) for 10, 20, or 30 min (**B**) or for 30 min (**C**) at 37 °C. The reaction products were separated by SDS-PAGE, and produced ubiquitin oligomers (Ub2, Ub3) and mono-ubiquitin (Ub1) were detected by silver staining. Arrows indicate the Flag-USP25 proteins.

We next examined the isopeptidase activity of USP25 toward ubiquitin chains. Flag-tagged WT and mutant forms of USP25 were expressed in HEK293T cells and immunoprecipitated with anti-Flag antibody. Precipitated proteins were eluted from the antibody with the Flag competing peptide. Coomassie staining of aliquots of the eluted proteins after SDS-polyacrylamide gel electrophoresis (PAGE) led to the estimation that 2~3 μg of each protein were recovered from cells in a 90-mm dish (Fig. 3A).

We first examined whether WT USP25 exhibits isopeptidase activity toward Lys48- and Lys63-linked ubiquitin chains by incubating the immunopurified USP25 proteins (~ 50 ng; ~50 fmol) with each tetra-ubiquitin chain (0.5 μg; 14.7 pmol) at 37 °C. In this reaction, the molar ratio between the enzyme (USP25) and the substrate (tetra-ubiquitin) was approximately 1:300. After the incubation, the reaction products were separated by SDS-PAGE, and ubiquitin oligomers and mono-ubiquitin were detected by silver staining (Fig. 3B, see Supplementary Fig. S1H for quantification). When Lys48-linked tetra-ubiquitin was incubated with USP25^WT^, the tetramer mostly disappeared, and di- and mono-ubiquitins appeared within 10 min of incubation. At 30 min, the amounts of tetra-ubiquitin and di-ubiquitin were further reduced and that of mono-ubiquitin increased. When Lys63-linked tetra-ubiquitin was incubated with USP25^WT^, a substantial amount of tetra-ubiquitin still remained and tri-ubiquitin was also observed at 10 min of incubation. After 30 min, the tetramer disappeared, but the trimer still remained. These results suggested that USP25 preferentially disassembles Lys48-linked ubiquitin chains to Lys63 chains. For both types of ubiquitin chains, substantial amounts of di-ubiquitin remained uncleaved even after 30 min of incubation, suggesting that USP25 exhibits higher isopeptidase activity toward ubiquitin oligomers longer than di-ubiquitin.

### Tandem UIMs are required for full catalytic activity of USP25 toward ubiquitin chains

We next examined the requirement of the tandem UIMs for the catalytic activity of USP25 (Fig. 3C, Supplementary Fig. S1I). When incubated with Lys48-linked ubiquitin chains, both USP25^ΔUIM1^ and USP25^ΔUIM2^ exhibited reduced isopeptidase activity compared with WT USP25 as determined from the remaining levels of tetra- and tri-ubiquitins. USP25^ΔUIM1UIM2^ was less active than these mutants as determined from the higher levels of tetra- and tri-ubiquitins, and lower level of mono-ubiquitin. When incubated with Lys63-linked ubiquitin chains, USP25^ΔUIM2^ was as active as WT USP25. In contrast, the activity of USP25^ΔUIM1^ was as weak as that of USP25^ΔUIM1UIM2^, as determined by the higher levels of tetra- and tri-ubiquitins, and lower level of mono-ubiquitin. As a negative control, no activity was detected in the catalytically-inactive mutant USP25^CS^. These results suggested that the tandem UIMs are required for the full catalytic activity of USP25 toward both Lys48- and Lys 63-linked ubiquitin chains.

### Conversion of ubiquitin chain-binding preference of USP25 by replacing the tandem UIM region

**Figure 4:**
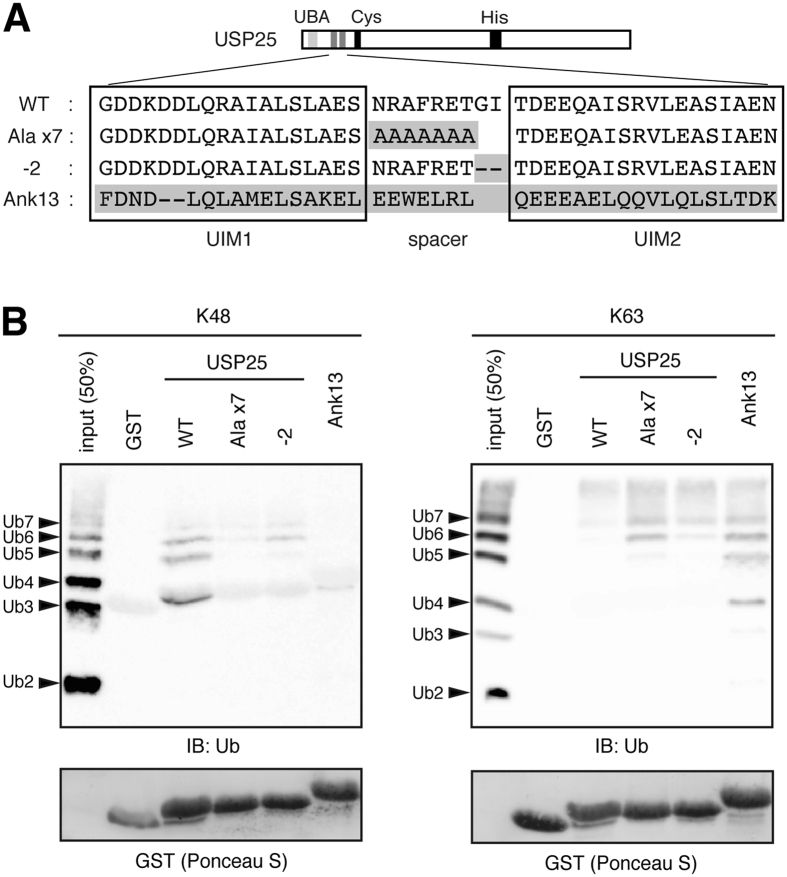
Conversion of ubiquitin chain-binding preference of USP25 by replacing the tandem UIM region. (**A**) Mutations introduced into the tandem UIM region of USP25 are shown. Amino acids highlighted in gray are different from those in USP25. (**B**) The tandem UIM regions of the USP25 mutants in (**A**) were fused to GST, immobilized on glutathione beads, and incubated with Lys48- or Lys63-linked ubiquitin oligomers (dimer-heptamer). Bound ubiquitin oligomers were detected by immunoblotting with anti-ubiquitin antibody (top). The amounts of GST-fusion proteins used in the experiments were assessed by staining with Ponceau S (bottom).

Preferential binding of the tandem UIMs of USP25 to Lys48-linked ubiquitin chains and the preferential isopeptidase activity of USP25 toward the same ubiquitin chain type raised the possibility that the ubiquitin chain substrate specificity of USP25 is determined by the ubiquitin chain binding specificity of its tandem UIMs. To test this possibility, we introduced several mutations into the tandem UIM region of USP25 in order to convert its ubiquitin chain-binding preference. Previous reports have demonstrated that the number of amino acids between two UIMs plays a critical role in determining the binding specificity of tandem UIMs toward Lys63-linked ubiquitin chains^20^,^24^. As the binding affinity of tandem UIMs toward Lys63 chains reaches the maximum level when two UIMs are spaced by seven amino acids, we replaced the 9-amino-acid spacer sequence between the UIM1 and UIM2 of USP25 (NRAFRETGI) with either a stretch of seven Ala residues (AAAAAAA, referred to as the Ala x7 mutation) or the same spacer sequence lacking the last two amino acids (NRAFRET, referred to as the -2 mutation) (Fig. 4A). We also replaced the entire tandem UIM region, including the spacer sequence, with the most C-terminally-located two tandem UIM region (UIM3, UIM4, and the 7-amino-acid spacer sequence between them) of Ankrd13A protein, which exhibits the binding specificity toward Lys63-linked ubiquitin chains^21^ (Fig. 4A, referred to as the Ank13 mutation).

We fused the tandem UIM region of these USP25 mutants to GST and examined their ubiquitin chain binding by the pull-down experiment. As expected, in all the three mutants, the affinity for Lys48-linked ubiquitin chains was reduced while the affinity for Lys63 chains was elevated, indicating that these mutations indeed resulted in the conversion in ubiquitin chain binding preference (Fig. 4B, Supplementary Fig. S1J).

### Ubiquitin chain-binding preference of tandem UIMs determines ubiquitin chain substrate preference of USP25

**Figure 5:**
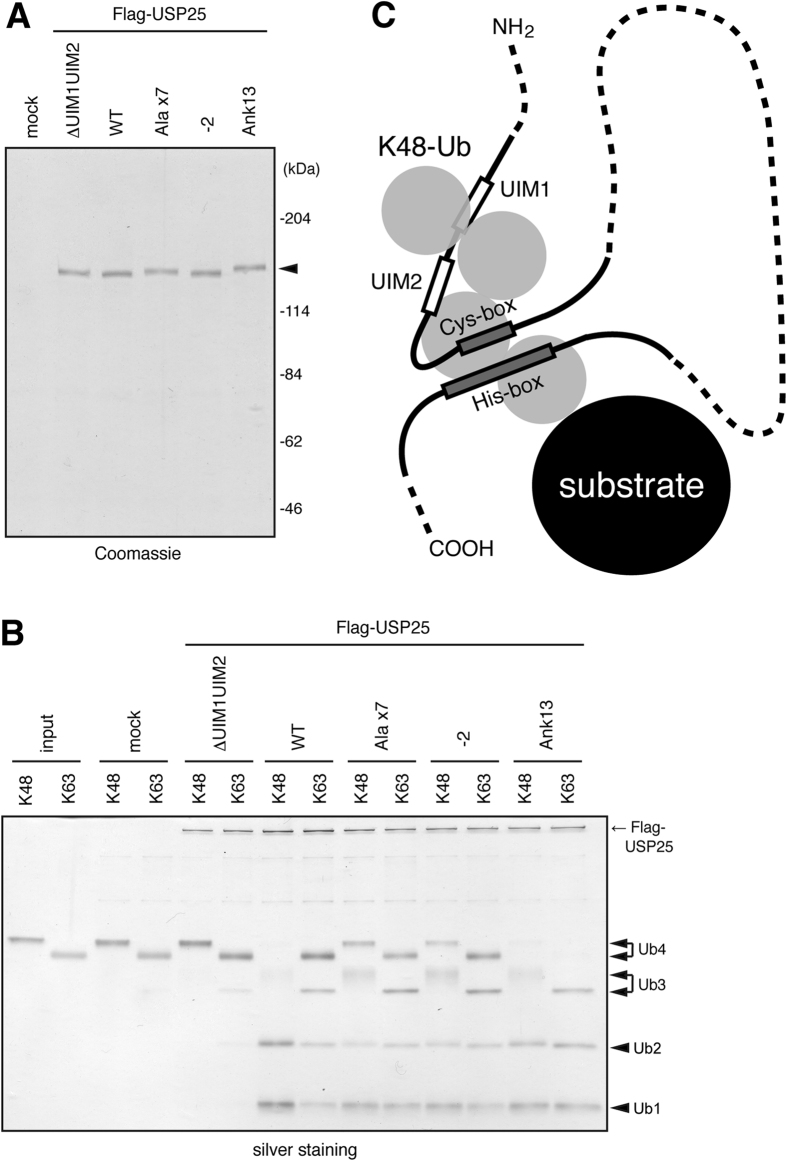
Ubiquitin chain-binding preference of UIMs determines ubiquitin chain substrate preference of USP25 catalytic activity. (**A**) Full-length WT USP25 and its mutants indicated in Fig. 4A were immunopurified from transfected HEK293T cells and detected by Coomassie staining as in Fig. 3A. (**B**) USP25 proteins prepared in (**A**) were incubated with Lys48- or Lys63-linked tetra-ubiquitin (Ub4) for 10 min at 37 °C. The reaction products were separated by SDS-PAGE, and ubiquitin oligomers (Ub2, Ub3) and mono-ubiquitin (Ub1) were detected by silver staining. (**C**) Schematic model for the role of tandem UIMs in USP25. By preferentially binding to the Lys48-linked poly-ubiquitin moiety, the tandem UIMs in USP25 hold Lys48 polyubiquitinated substrate proteins in close proximity to the catalytic core (Cys- and His-boxes), thereby conferring the substrate preference toward Lys48 polyubiquitinated proteins.

To examine the substrate preference of the USP25 mutants harboring the replaced UIM regions, we expressed their Flag-tagged versions in the full-length context in HEK293T cells and immunopurified them with anti-Flag antibody (Fig. 5A). The Ank13 mutant, which exhibited the highest affinity toward Lys63-linked ubiquitin chains (Fig. 4B), acquired the highest isopeptidase activity toward Lys63-linked ubiquitin chains as determined from the reduced and increased levels of tetra- and mono-ubiquitins, respectively (Fig. 5B, Supplementary Fig. S1K). The Ala x7 and -2 mutants also acquired higher catalytic activity toward Lys63 chains than WT USP25 as determined from the slightly reduced and increased levels of tetra- and tri-ubiquitins, respectively (Fig. 5B, Supplementary Fig. S1K). The lower activity of the Ala x7 and -2 mutants than that of the Ank13 mutant was consistent with their lower Lys63 ubiquitin chain-binding affinity than the Ank13 mutant (Fig. 4B). In contrast, the isopeptidase activity toward Lys48-linked ubiquitin chains was reduced in all three mutants, especially Ala x7 and -2 mutants, as determined from the increased levels of tetra- and tri-ubiquitins as well as the reduced levels of di- and mono-ubiquitins (Fig. 5B, Supplementary Fig. S1K).

## Discussion

USP25 has been shown to undergo sumoylation at a specific Lys residue in the tandem UIM region, and this modification inhibits the ubiquitin binding of the UIMs and suppresses the catalytic activity of USP25^25^. On the other hand, ubiquitination of the same Lys residue is demonstrated to elevate the catalytic activity of USP25^26^. However, the precise role of the tandem UIMs in USP25 has remained unclear. Our results in this study show that the UIMs play an essential role in Lys48 ubiquitin chain binding, leading to preferential isopeptidase activity of USP25 toward Lys48 ubiquitin chains.

UIMs are sometimes found in tandem and divided by spacer sequences of up to 10 amino acids. In proteins such as Rap80^20^,^24^, ataxin-3^27^, Ankrd13^21^, and USP37^22^, the tandem UIMs play a cooperative, rather than an additive, role in ubiquitin binding. Also in USP25, the ubiquitin-binding ability toward cellular ubiquitin-protein conjugates (Fig. 1) and purified ubiquitin oligomers (Fig. 2) was significantly reduced when either of the two UIMs was inactivated. In addition, also similarly to UIMs in other proteins, those in USP25 exhibited significantly higher affinity toward ubiquitin oligomers longer than trimer (Fig. 2).

Ubiquitin oligomer pull-down experiments with the tandem UIM region of USP25 showed that the UIMs preferentially bind to Lys48-linked ubiquitin chains. Co-immunoprecipitation experiments in cells expressing the K48R or K63R mutant of ubiquitin demonstrated that USP25 binds more effectively to Lys48-, than to Lys63-, polyubiquitinated proteins also in the cell. Previous studies have shown that several UIMs, such as those in Hrs^28^, Rap80^20^,^24^, and Ankrd13^21^, bind to Lys63-linked ubiquitin chains specifically, while others, such as those in ataxin-3^7^ and USP37^22^, bind to Lys48- and Lys63-linked chains with similar affinity. In contrast, reports of UIMs with binding specificity/preference to Lys48 ubiquitin chains have been limited. A single UIM in a yeast transcription factor Met4 was shown to bind preferentially to Lys48-linked ubiquitin chains, thereby regulating its proteasomal degradation^29^. Recently, tandem UIMs, divided by an 8-amino-acid spacer sequence, in a proteasome-associated protein AIRAPL were reported to bind Lys48-linked ubiquitin chains selectively^30^. In this protein, one of the UIMs is a double-sided UIM capable of binding two ubiquitin molecules simultaneously^31^. An X-ray crystallographic study showed that this double-sided UIM confers the specificity to Lys48-linked ubiquitin chains by binding to Lys48-linked two ubiquitin molecules winding around the UIM α-helix. As the UIM1 of USP25 has an amino acid sequence conserved in double-sided UIMs^30^, it is possible that the USP25 UIM1 also serves as a double-sided UIM which selectively binds to Lys48-linked ubiquitin chains. However, the double-sided UIM in an endosomal sorting protein Hrs^31^ exhibits preferential binding to Lys63-linked ubiquitin chains over Lys48 chains^28^, indicating that the double-sided feature of ubiquitin binding of UIMs does not necessarily define the binding specificity toward Lys48 chains. A structural-biology study of USP25 in complex with a Lys48-linked ubiquitin chain is therefore necessary to understand the basis of Lys48 chain preference of ubiquitin binding of USP25 at atomic levels.

Introduction of inactivating mutations into the tandem UIMs in USP25 resulted in reduced catalytic activity toward ubiquitin chains (Fig. 3). These results suggest that as proposed for other UIM-bearing DUBs, such as OTUD1^8^ and USP37^22^, the UIMs in USP25 play a role in elevating its catalytic activity. The ubiquitin chain substrate preference of the USP25 isopeptidase activity has been unclear. Purified USP25 disassembles both Lys48- and Lys63-linked ubiquitin chains^32^. In cells, USP25 has been reported to remove Lys48 chains from TRAF3^16^,^17^, and Lys63 chains from TRAF5/6^16^,^33^, selectively. Upon viral infection, USP25 removes unidentified ubiquitin chain types from TRAF6 and thereby inhibits its degradation by autophagy^17^. In this study, we show that USP25 preferentially disassembles Lys48-linked ubiquitin chains to Lys63 chains (Fig. 3). Together with that the UIMs in USP25 exhibit binding preference toward Lys48-linked ubiquitin chains (Fig. 2), these results suggest that the tandem UIMs not only elevate the catalytic activity, but also confer ubiquitin chain preference to the isopeptidase activity, of USP25, by selectively holding Lys48-linked ubiquitin chains in the proximity of the catalytic core. In strong support of this possibility, when the amino acid sequence of the spacer region between the two UIMs of USP25 was mutated to convert the binding preference to Lys63 ubiquitin chains or when the tandem UIM region was replaced by that of Ankrd13A protein with Lys63 ubiquitin chain-binding specificity, the USP25 mutants acquired substrate preference toward Lys63-linked ubiquitin chains (Figs 4 and 5). Again, a structural-biology study of USP25 complexed with a Lys48-linked ubiquitin chain is important to verify this model on the role of the UIMs in USP25 (Fig. 5C).

Six of ~90 human DUBs bear UIMs^3^. For some of them, the role of UIMs in determining the ubiquitin chain substrate specificity has been revealed. Ataxin-3, a DUB of the ataxin-3/Josephin family, contains three UIMs, two of which are positioned in tandem. Ataxin-3 selectively disassembles Lys63-linked ubiquitin chains, but loses the specificity and equally disassembles Lys48 and Lys63 chains when all the three UIMs are inactivated by mutations, suggesting that the UIMs play an important role in rendering the substrate specificity toward Lys63-linked ubiquitin chains^7^. The precise role for the UIMs in this regulation, however, is unclear because they equally bind to Lys48- and Lys63-linked ubiquitin chains^7^. In the case of OTUD1, an OTU family DUB with a single UIM located just C-terminal to the catalytic domain, the catalytic activity as well as the substrate specificity toward Lys63 ubiquitin chains are significantly reduced when the UIM is deleted^8^. These results suggest a role for the UIM in increasing the Lys63 chain-specific ubiquitin isopeptidase activity of OTUD1, although the ubiquitin chain-binding specificity of the OTUD1 UIM has not been reported. Compared to the role of these UIMs in determining the Lys63 ubiquitin chain substrate specificity of DUBs, involvement of ubiquitin-binding domains in regulating the substrate specificity of DUBs toward Lys48 ubiquitin chains has been unknown. The results in this study provide the first example of DUBs in which the binding preference of a ubiquitin-binding domain toward Lys48 ubiquitin chains determines the preference of its isopeptidase activity toward Lys48 ubiquitin chains.

## Materials and Methods

### cDNA expression constructs

The cDNA for human USP25 was provided by the RIKEN BioResource Center (Tsukuba, Japan) and inserted into the N-terminally Flag epitope-tagged mammalian expression vector pME-Flag^34^. Introduction of mutations into the USP25 cDNA was performed using the QuikChange site-directed mutagenesis system (Stratagene, La Jolla, CA, USA) or PrimeSTAR Mutagenesis Basal Kit (Takara, Tokyo, Japan). The region of USP25 containing the tandem UIMs (amino acids 94–144), with or without mutations, was inserted into pGEX 6P-2 (GE Healthcare, Little Chalfont, UK) to construct expression vectors for the GST-UIM fusion proteins. Expression vectors for GST-fused UIM regions of Ankrd13A (UIM3 and UIM4) and USP37 (UIM2 and UIM3) were constructed as described previously^21^,^22^. Expression vectors for HA-tagged K48R and K63R mutants of ubiquitin were constructed by introducing point mutations into the HA-ubiquitin expression vector pcDNA3.1-HA-Ub (provided by Dr. Suzuki, Tokyo Metropolitan Institute of Medical Science, Tokyo, Japan) using the QuikChange site-directed mutagenesis system (Stratagene).

### Cell culture and DNA transfection

HEK293T cells were grown in Dulbecco’s modified Eagle’s medium supplemented with 10% fetal bovine serum (FBS). DNA transfection was performed using the polyethylenimine transfection reagents (Polyscience, Warrington, PA, USA) following the manufacturer’s instructions.

### Immunoprecipitation and immunoblotting

HEK293T cells were lysed in 20 mM Tris-HCl, pH 7.4, 100 mM NaCl, 50 mM NaF, 0.5% Nonidet P-40, 1 mM EDTA, 1 mM phenylmethylsulfonyl fluoride, 1 μg/ml aprotinin, 1 μg/ml leupeptin, and 1 μg/ml pepstatin A, and the supernatants were collected after centrifugation. Immunoprecipitation and immunoblotting were performed using standard procedures. Anti-Flag antibody (1 μg; clone M2, Sigma-Aldrich, St. Louis, MO, USA) was used for immunoprecipitation. Primary antibodies for immunoblotting were: anti-ubiquitin (1:1,000; clone P4D1, Cell Signaling Technology, Danvers, MA, USA), anti-Flag (1 μg/ml; clone M2), and anti-α-tubulin (1:2,500; Abcam, Cambridge, MA, USA) antibodies. Secondary antibodies were peroxidase-conjugated anti-mouse IgG and anti-rabbit IgG antibodies (GE Healthcare). Blots were detected using ECL Western Blotting Detection Reagents (GE Healthcare) and the ImageQuant LAS 4000 mini chemiluminescence detection system (GE Healthcare).

### Ubiquitin pull-down assay

GST-UIM fusion proteins were purified from transformed *E. coli* cells using glutathione-Sepharose beads (GE Healthcare). Each fusion protein (4 μg) was immobilized on glutathione-Sepharose beads (10 μl) and incubated with different concentrations of Lys48- or Lys63-linked tetra-ubiquitin chain (Boston Biochem, Boston, MA, USA), or Lys48- (1 μg) or Lys63- (0.2 μg) linked ubiquitin oligomers (dimer-heptamer, Boston Biochem) in 20 mM Tris-HCl, pH 8.0, 50 mM NaCl, 50 mM NaF, and 0.5% Nonidet P-40 for 16 h at 4 °C. The beads were washed with the same buffer, and bound ubiquitin oligomers were detected by immunoblotting after SDS-PAGE.

Flag-tagged full-length USP25 proteins were purified from transfected HEK293T cells. Transfected cells were lysed in 20 mM Tris-HCl, pH 7.4, 150 mM NaCl, 1 mM EDTA, 0.5% Nonidet P-40, and 1% SDS for 10 min at 100 °C (hot-lysis). After centrifugation, supernatants were diluted fivefold with the “hot lysis” buffer lacking SDS and used for immunoprecipitation with agarose beads conjugated with anti-Flag antibody (anti-Flag M2 affinity gel, Sigma-Aldrich). Immunoprcipitated USP25 proteins, immobilized on the anti-Flag beads, were used for the ubiquitin pull-down assay as described above for GST-UIM fusion proteins.

### DUB assay

Flag-tagged USP25 proteins were expressed in HEK293T cells in a 90-mm dish, immunoprecipitated from their lysates using anti-Flag M2 affinity gel (Sigma-Aldrich), and eluted from the antibody by incubation with 100 μl of Tris-buffered saline containing the Flag peptide (400 μg/ml, Sigma-Aldrich) and 1 mM dithiothreitol. Purity and concentration of eluted USP25 proteins were assessed by Coomassie staining after SDS-PAGE using purified bovine serum albumin as a standard. Immunopurified USP25 proteins (~50 ng: ~50 fmol) were incubated with 0.5 μg (14.7 pmol) of Lys48- or Lys63-linked tetra-ubiquitin chain (Boston Biochem) in 6 μl of Tris-buffered saline containing 1 mM dithiothreitol at 37 °C. Reaction products were separated by SDS-PAGE and detected by silver staining.

### Quantification of the intensity of protein bands/smear detected by immunoblotting or silver staining

The intensity of bands of ubiquitin chains and smear of ubiquitinated proteins, detected by immunoblotting or silver staining after SDS-PAGE, were quantified using the NIH image analysis program ImageJ.

## Additional Information

**How to cite this article**: Kawaguchi, K. *et al*. Tandem UIMs confer Lys48 ubiquitin chain substrate preference to deubiquitinase USP25. *Sci. Rep.* **7**, 45037; doi: 10.1038/srep45037 (2017).

**Publisher's note:** Springer Nature remains neutral with regard to jurisdictional claims in published maps and institutional affiliations.

## Supplementary Material

Supplementary Information
