# Supplementary material for: Tandem UIMs confer Lys48 ubiquitin chain substrate preference to deubiquitinase USP25
Source: Sci Rep. 2017 Mar 22;7:45037. doi: 10.1038/srep45037 (PMC5361193; doi:10.1038/srep45037)

Supplementary Information

# Tandem UIMs confer Lys48 ubiquitin chain substrate preference to deubiquitinase USP25

**Kohei Kawaguchi[1,2], Kazune Uo[2], Toshiaki Tanaka[2] & Masayuki Komada[1,2]**

[1]Cell Biology Unit, Institute of Innovative Research, Tokyo Institute of Technology, Yokohama 226-8501, Japan; [2]School of Life Science and Technology, Tokyo Institute of Technology, Yokohama 226-8501, Japan

**Supplementary Figure S1. Quantification of the intensity of protein bands/smear detected by immunoblotting or silver staining**

The intensity of bands of ubiquitin chains and smear of ubiquitin-protein conjugates, detected by immunoblotting or silver staining after SDS-PAGE, were quantified using NIH ImageJ. The intensity of control areas with the same size was subtracted from that of each sample area.

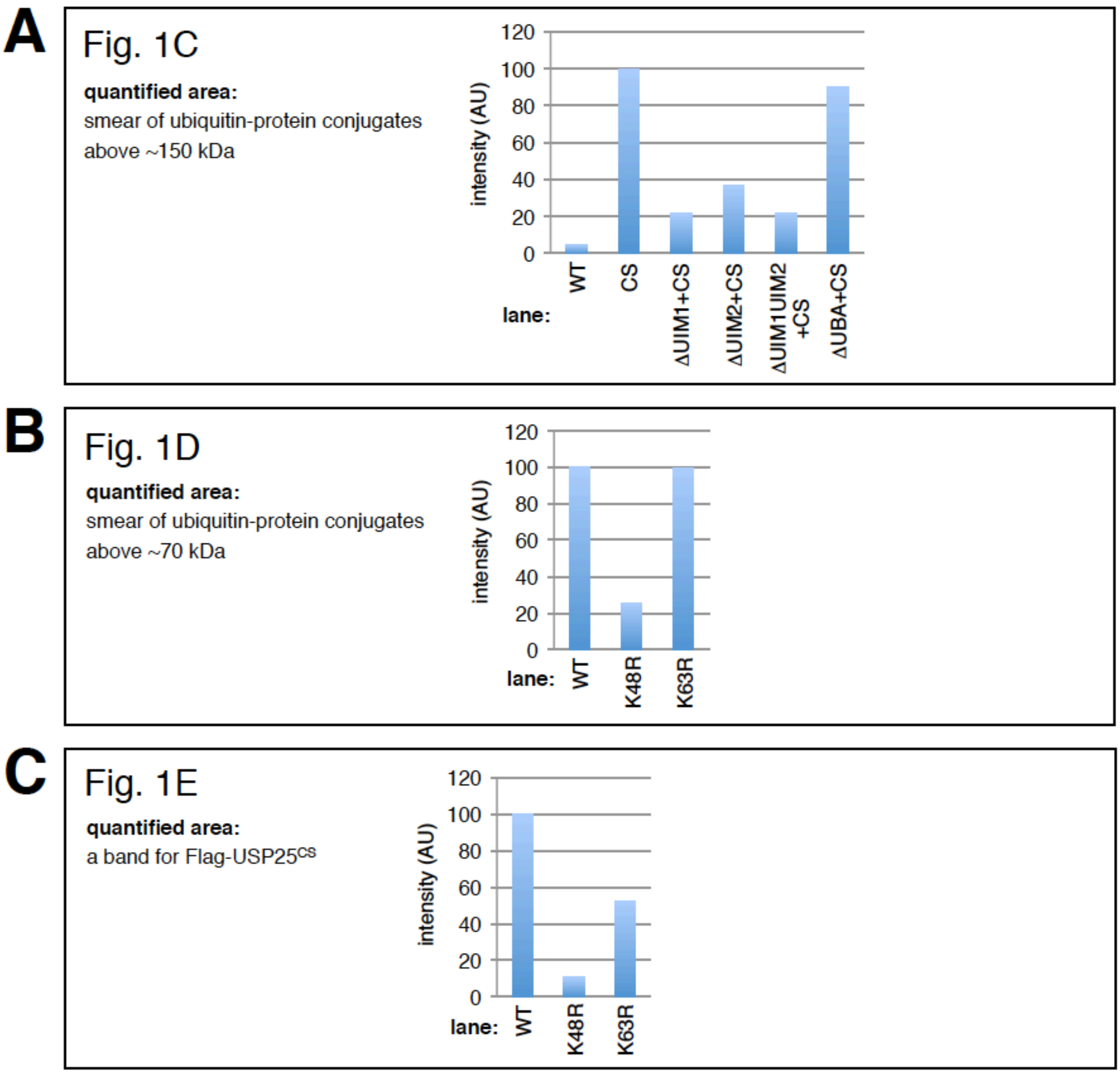

**D**

## Fig. 2B

**quantified area:**
a band for tetra-ubiquitin

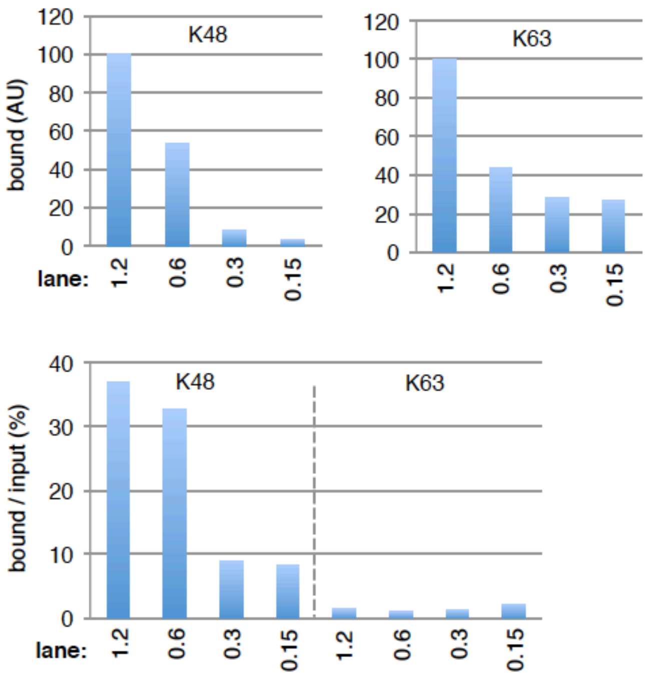


**E**

## Fig. 2C

**quantified area:**
bands for ubiquitin oligomers
(trimer-heptamer)

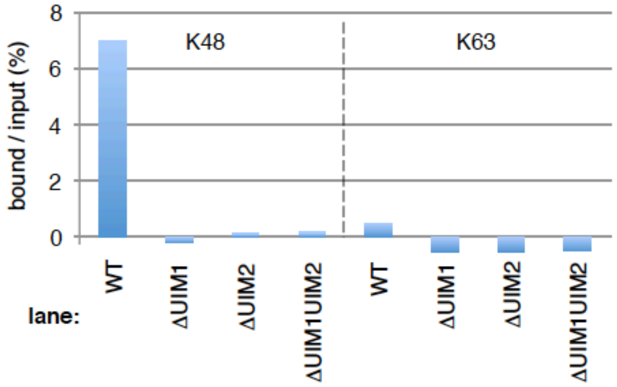


**F**

## Fig. 2D

**quantified area:**
bands for ubiquitin oligomers
(tetramer-heptamer)

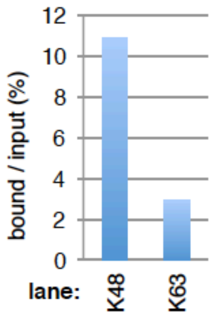

**G**

## Fig. 2E

**quantified area:**
bands for ubiquitin oligomers (tetramer-heptamer)

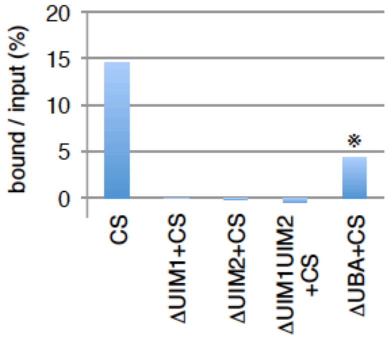


※ reduced ubiquitin binding to USP25$^{\Delta UBA+CS}$ is due to reduced amount of USP25$^{\Delta UBA+CS}$ used for pull-down.

**H**

## Fig. 3B

**quantified area:**
bands for tetra- and tri-ubiquitin

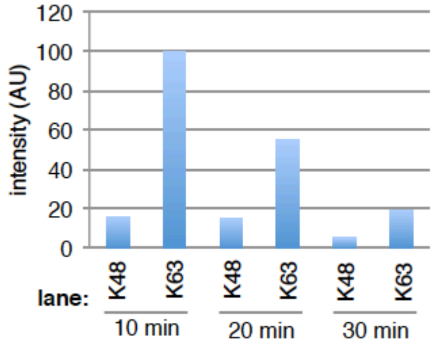


**I**

## Fig. 3C

**quantified area:**
bands for tetra- and tri-ubiquitin

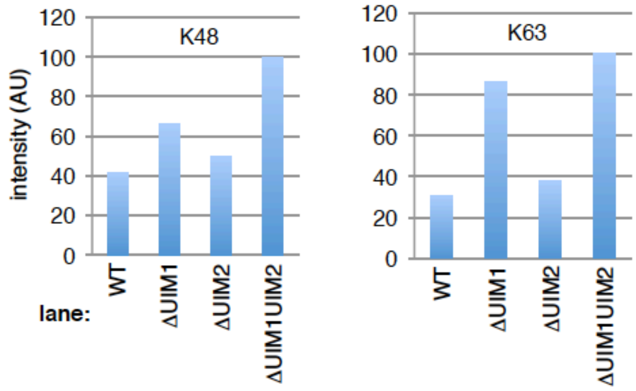


**J**

## Fig. 4B

**quantified area:**
bands for ubiquitin oligomers (trimer-heptamer)

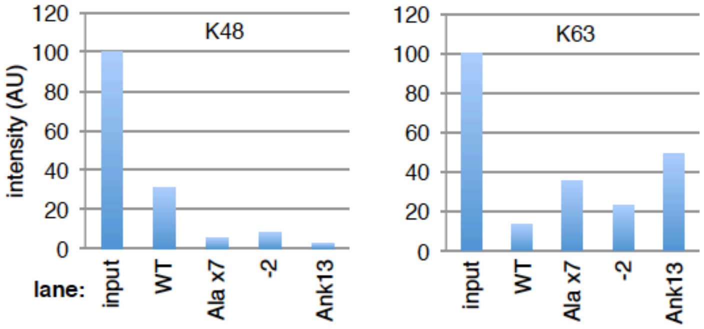


**K**

## Fig. 5B

**quantified area:**
bands for tetra- and tri-ubiquitin

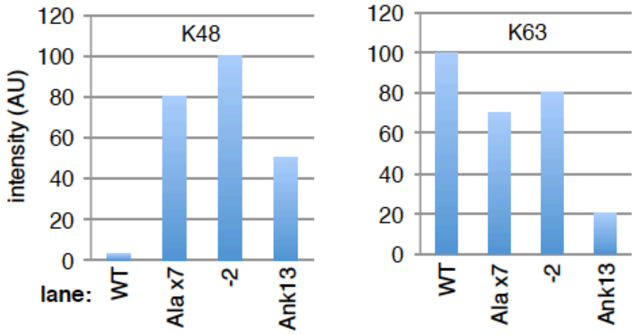

Supplement: Supplementary Information [file srep45037-s1.pdf]
